# Alternation of brain intrinsic activity in patients with hypertensive retinopathy: a resting-state fMRI study

**DOI:** 10.18632/aging.203510

**Published:** 2021-09-13

**Authors:** Jin-Yu Hu, Hui-Ye Shu, Qiu-Yu Li, Shi-Nan Wu, Rong-Bin Liang, Qian-Min Ge, Li-Juan Zhang, Yi-Cong Pan, Yi Shao

**Affiliations:** 1 Department of Ophthalmology, The First Affiliated Hospital of Nanchang University, Jiangxi Province Ocular Disease Clinical Research Center, Nanchang 330006, Jiangxi, People’s Republic of China; 2 Hunan University of Medicine, Huaihua 418000, Hunan, People’s Republic of China

**Keywords:** hypertensive retinopathy, functional MRI, ALFF, spontaneous brain activity, resting-state

## Abstract

Objective: To investigate the changes of amplitude of low-frequency fluctuation (ALFF) in brain regions of patients with hypertensive retinopathy by using resting-state functional magnetic resonance imaging (rs-fMRI) and change in the relationship of ALFF value with potential emotional and psychological changes.

Methods: Thirty-one patients with hypertensive retinopathy (HR) (16 men and 15 women) and 31 healthy controls (HCs; 16 men and 15 women) matched for age, sex, and weight were enrolled in the research. The changes in mean ALFF values could reflect brain activity between HR patients and HCs. We used the independent samples t-test to evaluate different demographic and general information between the two groups. Two-sample t-test was used to detect differences of mean ALFF values in the brain region between the two groups using the same software.

Results: The ALFF values in the brain areas of HR and HCs were different. HR patients had lower ALFF value in the left medial superior frontal gyrus and left middle frontal gyrus than the HCs. The higher ALFF values were found in the cerebellum (left inferior and right superior lobes, vermis) and left inferior temporal gyrus of the HR patients than the controls.

Conclusion: Our findings showed fluctuations in ALFF values in the HR patients’ brain regions. ALFF values reflect over or reduced activity in brain regions. Abnormal ALFF values in these brain areas can predict early HR development, preventing the malignant transformation of hypertensive microangiopathy.

## INTRODUCTION

**Figure 1:**
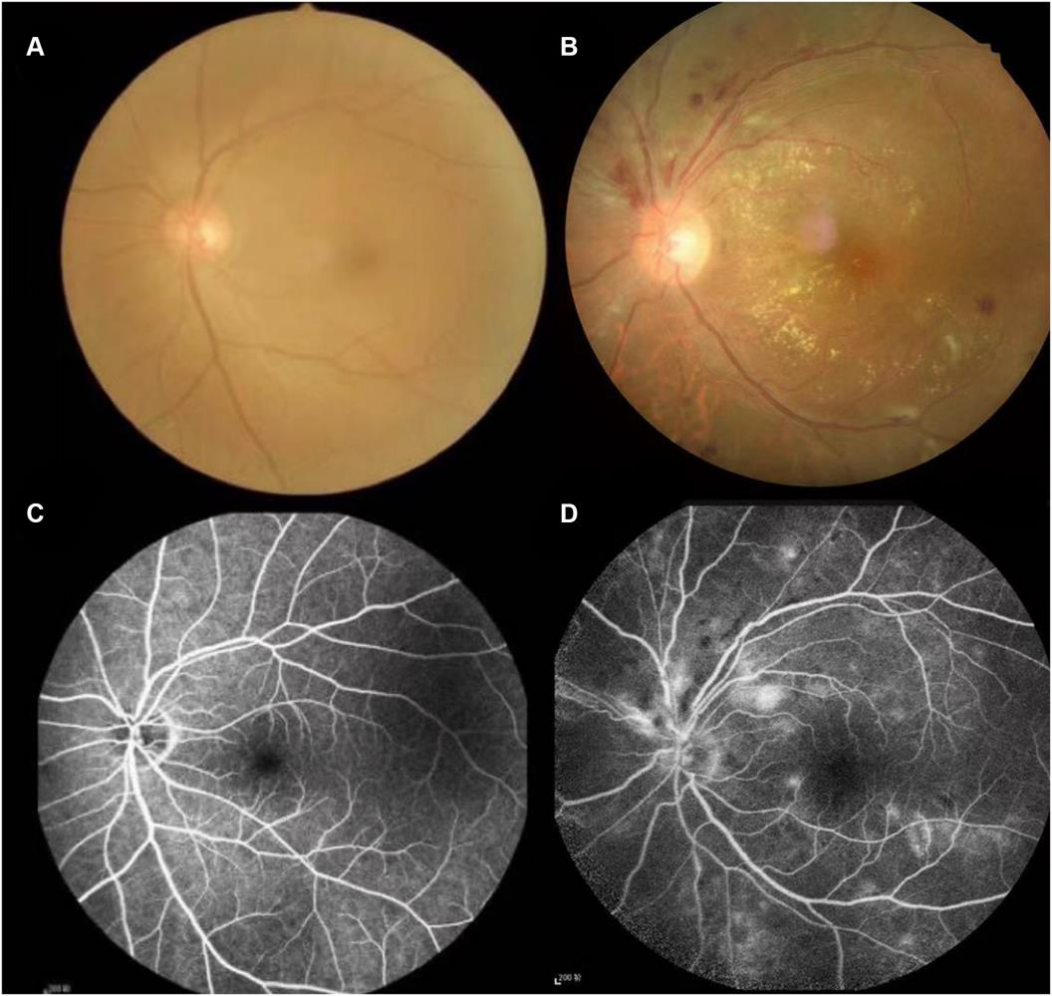
**Typical examples of fundus camera and fluorescence fundus angiography in healthy people and patients with Hypertensive retinopathy.** (**A** and **C**) of Figure 1 shows the left retinal fundus photos and corresponding fluorescence fundus angiography of healthy people. (**B** and **D**) of Figure 1 shows the left retinal fundus photos and fluorescence fundus angiography of patients with hypertensive retinopathy. Retinal arteriosclerosis, stenosis, wall light reflection enhancement, silver filiform, artery and vein cross compression phenomenon, the distal vein and capillary dilatation, retinal edema, bleeding and exudation.

Hypertension is the most common cardiovascular disease and can cause atherosclerosis, resulting in narrowing of the arterial lumen, thereby hindering blood circulation [1]. In the early stages of hypertension, the eye’s fundus is often normal. When the blood pressure continues to rise, it can cause systemic arteriosclerosis, which occurs in patients with retinopathy, known as hypertensive retinopathy [2]. The degree of hypertensive retinopathy is positively correlated with the degree of systemic major organ disease and is a clinical manifestation that precedes all other complications. More than 70 percent of patients with primary hypertension have different degrees of retinopathy, [3] depending on age, degree of blood pressure elevation, and disease duration. Studies have shown that patients with hypertension of <5 years have a significantly increased risk of retinopathy. The fundus changes in hypertension are mainly related to the degree of increased blood pressure [4]. The shift in fundus is aggravated with the increase of systolic and diastolic blood pressure, which is more closely related to the rise in diastolic blood pressure [4]. Fundus photography and fluorescence imaging may show typical signs of hemorrhage, lipid exudation, and fluorescein leakage, and in severe cases, may show optic papilledema [5] (Figure 1). Hypertensive retinopathy (HR) is the ocular manifestation of systemic vascular disease caused by hypertension. Fundus retinal blood vessels belong to the category of microvessels, which is the only blood vessel that can be directly visualized [6]. It is clinically essential for estimating the course of the disease, its severity, and prognosis.

Functional magnetic resonance imaging (fMRI) for localization of brain function is a very effective and non-invasive technique for studying fluctuations in functionally related brain areas and has become widely used to study brain function [7]. Although it is a non-invasive technique, it can accurately and reliably locate specific cortical areas of cerebral activity with a spatial resolution of 2 mm. FMRI can track signal changes in real time with a temporal resolution of 1 second, during which the participant's unique brain activation areas can be quickly assessed before identifying functional brain connections. It can be repeated in a variety of ways to scan the subject. FMRI data can be used as a preliminary reflection of mental representations and information processing [8]. Briefly, fMRI technology can display slight changes in MR signals caused by blood oxygenation in venous capillaries in various brain regions. The functional localization of brain activity can be realized on a standard living body without being damaged. As a new non-invasive method, rs-fMRI can be more comfortably implemented clinically. The continuous development of rs-fMRI technology and analytical methods have provided a way for in-depth exploration of pathophysiological mechanisms of nervous system diseases. Clinicians can detect brain abnormalities as early as possible by assessing the function of regional and neural circuits in HR patients’ brains in the resting state.

Amplitude of low-frequency fluctuation (ALFF) reflects the average intensity of the signal in the low-frequency part of each voxel in the blood-oxygen-level-dependent (BOLD) signal. Generally, low frequency refers to 0.01–0.08 Hz. The amplitude can represent the activity intensity of the brain. The BOLD signal intensity can reflect the activation and deactivation of the brain. Each voxel in the brain can calculate an ALFF value, and each ALFF value represents the activity intensity of the voxel in the brain. The ALFF is an effective fMRI analytic technique to evaluate the brain regions’ resting-state activity [9]. Existing research shows that the excellent performance of ALFF is mainly dependent on its test-retest reliability [10–11]. The ALFF value is used to apply rs-fMRI data for disease characterization, depending on its general computability and reliability [12]. In recent years, the application of ALFF technology has revealed spontaneous abnormal activity of local cerebral regions in ophthalmic diseases. The similarity between the functional connection mode in the resting state and the task state’s activation mode is nearly 80%. The resting state’s functional connection is used to explore individual differences in cognitive functions and attention and explore individual differences in complex social decisions [13]. Thus, using this technology, we can easily explain the pathophysiological mechanism of these diseases from different dimensions, which can provide brand-new ideas for guiding the diagnosis, treatment, and evaluation of disease prognosis.

Most ophthalmic studies of diseases use ALFF to analyze the correlation between intrinsic brain activities and mental diseases, such as primary angle-closure glaucoma (PACG), late monocular blindness (MB), adult comitant exotropia strabismus (CES), acute unilateral open globe injury (OGI), and high myopia [14–18] (Table 1). Because of the scarcity of HR patients’ clinical data, previous studies have not extended to HR. To address this issue, we investigated functional changes in the brain network and their relationship with HR patients’ behavioral performance by using rs-fMRI and ALFF technology.

**Table 1:** ALFF method applied in ophthalmology-related diseases.

**Authors**	**Year**	**Diseases**
Huang et al. [14]	2015	Primary angle-closure glaucoma
Li et al. [15]	2016	Late monocular blindness
Tan et al. [16]	2016	Adult comitant exotropia strabismus
Tan et al. [17]	2016	Acute unilateral open globe injury
Yu et al. [18]	2020	Myopia before and after Lasik surgery

Abbreviation: ALFF: the amplitude of low-frequency fluctuation.

## METHODS

### Subjects

From August 2018 to October 2019, we recruited 31 patients with HR (16 men and 15 women) from the Ophthalmology Department of the First Affiliated Hospital of Nanchang University. The criteria for diagnosing HR were as follows: 1) fundus examination performed based on a systematic diagnosis of stage 2 or above hypertension (systolic blood pressure ≥140 mmHg or diastolic blood pressure ≥90 mmHg); and 2) a record of microaneurysms, retinal hemorrhage, cotton thread spots, exudate, arteriovenous crossing, arteriolar stenosis, and papilledema, which were used to classify retinopathy. Retinopathy was graded according to the Keith–Wagener classification [19] as follows: Grade I, mild retinal artery stenosis or sclerosis; Grade II, moderate arterial stenosis with arteriovenous crossing; Grade III, arterial stenosis and large arteriovenous crossover changes accompanied by bleeding, exudates, and cotton spots; and Grade IV, severe grade III with papilledema. The exclusion criteria for HR patients were as follows: 1) history of eye surgery, diabetes, and nervous system disease; 2) unable to undergo MRI scanning for subjective and objective reasons; 3) alcohol intake >30 g/day; and 4) old or multiple cerebral infarctions in the MRI scan. We recruited 31 healthy age- and sex-matched volunteers (16 males and 15 females) from various communities in Nanchang City, Jiangxi Province, China, as the healthy control (HC) group. All HCs fulfilled the following conditions: 1) no neurologic- or eye-related illness; 2) head MRI scan showing normal brain parenchyma, and 3) no contraindications for MRI. The Medical Ethics Committee of the First Affiliated Hospital of Nanchang University approved this study. The research method conforms to the tenets of the Declaration of Helsinki. All participants were aware of the research purposes, the procedure, and provided voluntary signed informed consent.

### Parameters

All MRI examinations were generated by a 3T MR scanner (Trio; Siemens, Munich, Germany). During the scanning, all participants were required to be in the supine position, keep awake, quiet, close their eyes, and not indulge in active thinking. Using a three-dimensional spoiled gradient recalled echo sequence to obtain T1-weighted images, the following specific scan parameters were used: For 176 structural image scans, the field of view was 250 × 250 mm; thickness, 1.0 mm; gap, 0.5 mm; repetition time, 1900 ms; echo time, 2.26 ms; flip angle, 9°; and acquisition matrix, 256 × 256. For the 240 functional image scans, the field of view was 220 × 220 mm; gap, 1.2 mm; thickness, 4.0 mm; echo time, 30 ms; repetition time, 2000 ms; flip angle, 90°; and acquisition matrix, 64 × 64. The scanning duration from start of completion was about 15 min.

### Data analysis of fMRI

fMRI data processing mainly includes two steps of raw data preprocessing and statistical analysis of image data. MRIcro software (http://www.mricro.com) was used to classify the available data to eliminate defective data. Owing to the magnetization equilibration, the first 10 volumes were abandoned to eliminate potential artifacts from scanner instability or the surrounding environment. The remaining data were all preprocessed using the DPARSFA (http://rfmri.org/DPARSF). Interlayer scanning is generally adopted for brain scanning. Given the large difference in scanning time of the same brain region, slice timing is required to correct these differences for scanning at the same time point. Rigid body transformation is used to fix the brain in all the images at the same Target. Because the subject’s head could move in the MRI machine, Realign was used to adjust the orientation for head movement correction. Exclude subjects with maximum displacement in the X, Y or Z plane exceeding 1.5-mm and angular motion exceeding 1.5 degrees. To reduce the error, the effects of head motion were cancelled out by Friston six head-motion parameters; the latest research shows that the high-order models have advantages in offsetting head motion [20–21].

Upon completing head motion correction, the next step was to co-register the structure image and function image. The functional and structural images were manually adjusted, including head rotation, translation, and origin positioning, to increase the accuracy of co-registration, segmentation, and normalization. Each person’s brain has individual differences, and hence it needs unified standardization to the same space before they can be compared with each other (voxel size default: 3 mm × 3 mm × 3 mm). The T1-weighted image was registered into the fMRI space and the Montreal Neurological Institute (MNI) standard template was used to convert the image into the MNI space.

Before statistical analysis, spatial smoothing was performed on the acquired data to eliminate artifacts, making the data more consistent with distributional assumptions. In accordance with the analysis requirements of gaussian kernel. There is a linear drift due to increased temperature or subject acclimation, the time sequence of each voxel must eliminate the linear drift [22]. Operate the machine to improve the accuracy of the functional diagram and reduce the influence of other factors. Resting fMRI signals filtered at low frequencies (0.01 ~ 0.08Hz) show significant physiological significance, reflecting spontaneous neural activity Previous studies have described in detail the process of calculating the value of ALFF [23]. Normalized ALFF was obtained by dividing ALFF by the average ALFF of all voxels in the whole brain.

### Mass control

Researchers who have received consistency training perform clinical scale scoring, and neither MRI nor scale evaluators know the grouping of patients. To ensure that the fMRI images conformed to the standards, the physician asks the subject to remain as calm as possible before the scan and to keep the body still during the scan. During scanning, the doctor observed the original image and removed or rescued any image with noticeable artefacts or deformation.

### Data analysis

Demographic and clinical variables of HR and HC groups were analyzed by SPSS v20.0 software (SPSS Inc, Chicago, IL, USA). The relevant data of the subjects were tested for normality. Differences in the HR and HC groups were evaluated with the independent samples *t*-test (statistical threshold with *P* < 0.05). The differences of average ALFF values in the brain region between the HR and HCs using the same software with the two-sample *t*-test; after Gaussian correction, the statistical significance threshold was set to *P* < 0.05. We analyzed different ALFF values in the two groups’ brain regions and speculated that differences in ALFF values could distinguish HR patients from HCs.

### Ethical statement

All research methods were approved by the committee of the medical ethics of the First Affiliated Hospital of Nanchang University and were in accordance with the 1964 Helsinki declaration and its later amendments or comparable ethical standards. All subjects were explained the purpose, method, potential risks and signed an informed consent form.

## RESULTS

### Characteristics of the study population

The clinical course of HR in patients was 1.66 ± 12.65 years. The HR and HC groups showed statistically significant differences in Best Corrected Visual Acuity (BCVA), diastolic blood pressure, and systolic blood pressure (*P* > 0.05). There was no noticeable difference in the following aspects: sex (*P* > 0.99), age (*P* = 0.756), weight (*P* = 0.804), dominant hand (*P* > 0.99), or heart rate (*P* = 0.067) (Table 2).

**Table 2:** Conditions of participants included in the study.

**Condition**	**HR**	**HCs**	***t***	*P*-value^*^
Male/female	16/15	16/15	N/A	>0.99
Age (years)	54.35 ± 6.87	51.36 ± 6.86	0.178	0.756
Weight (kg)	69.64 ± 4.42	65.57 ± 5.75	0.202	0.804
Handedness	31R	31R	N/A	>0.99
Duration of HR (years)	31.66 ± 12.65	N/A	N/A	N/A
Best-corrected VA-left eye	0.66 ± 0.17	1.05 ± 0.25	–3.764	0.007
Best-corrected VA-right eye	0.57 ± 0.21	1.10 ± 0.20	–3.835	0.003
Confrontation VF	Full	Full	–N/A	N/A
SBP (mmHg)	166 ± 25	129 ± 18	2.864	0.023
DBP (mmHg)	101 ± 15	76 ± 11	2.142	0.016
HR1 (beats per minute)	69 ± 12	62 ± 14	0.825	0.067
IOP (mmHg)	16.23 ± 3.12	15.69 ± 4.19	0.725	0.21

^*^p < 0.05 Independent *t*-tests comparing two groups, Data shown as mean ± standard deviation. Abbreviations: DBP: diastolic blood pressure; HR: Hypertensive retinopathy; HR1: heart rate; HCs: normal controls; VA: visual acuity; N/A: not applicable; SBP: systolic blood pressure; VF: Visual field; IOP: intra ocular pressure.

### ALFF Differences

**Figure 2:**
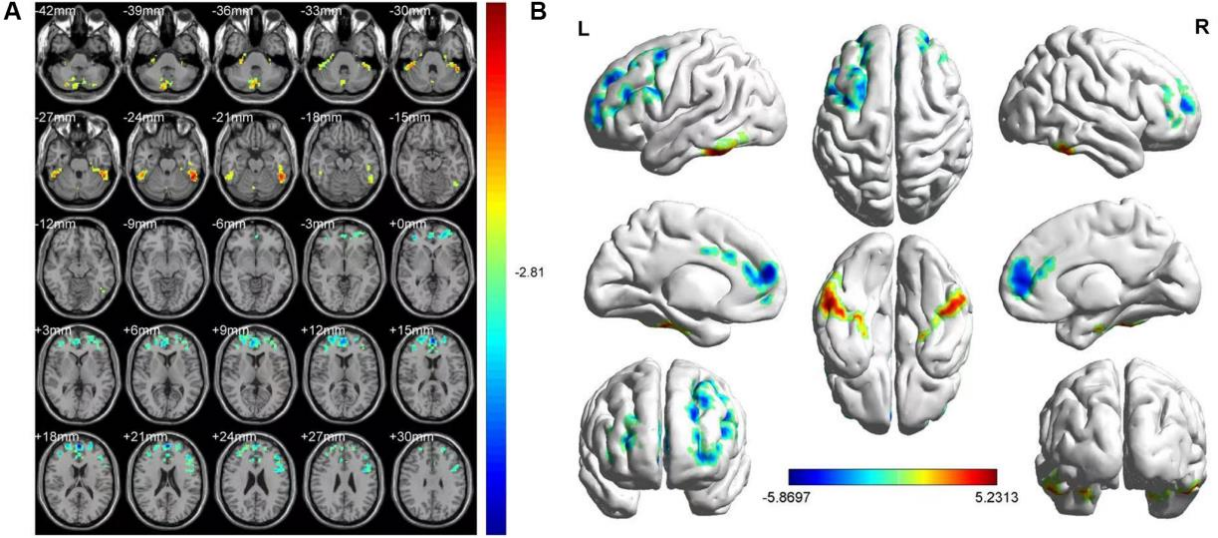
**FMRI showed brain regions where the ALFF values' difference was statistically significant between the HR and HC groups.** The difference of ALFF value is shown in (**A**, **B**) shows the ALFF changes in the cerebral cortex. Different is shown in the left medial SFG, left MFG, left ICL, vermis, left ITG and right SCL. The yellow and red areas represent an increase in ALFF values; the blue regions reduce ALFF values.

**Figure 3:**
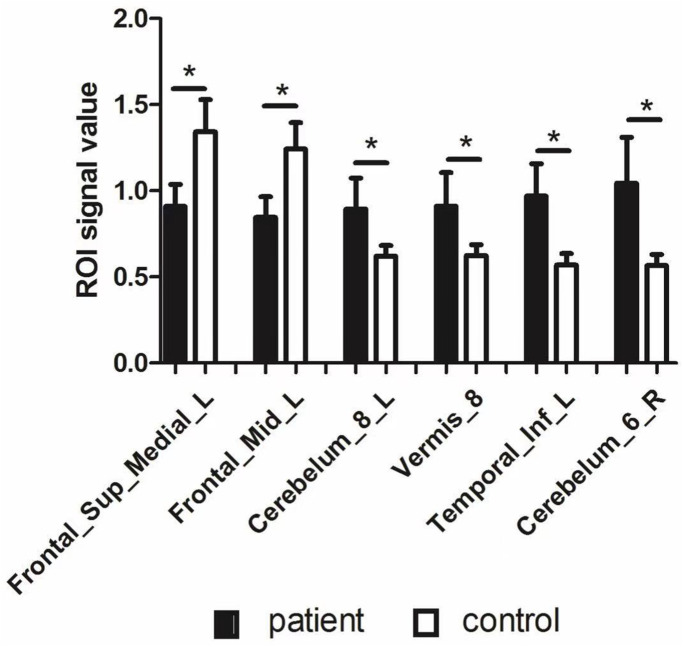
**The ROI signal value of brain areas between patients with HR and healthy control.** Abbreviations: ALFF: amplitude of low-frequency fluctuation; L: left; R: right; Frontal_Sup_Medial_L: left medial superior frontal gyrus; Frontal_Mid_L: left middle frontal gyrus; Temporal_Inf_L: left inferior temporal gyrus.

Upon comparing the HR and HC groups, HR patients’ ALFF was lower in the left medial superior frontal gyrus(LMSFG) and left middle frontal gyrus (LMFG) than that of the HCs (Figure 2A and 2B [blue]; Table 3). Higher ALFF values in the brain areas were the left inferior cerebellar lobe, cerebellar vermis, left inferior temporal gyrus (LITG), and right superior cerebellar lobe (Figure 2A and 2B [red and yellow]; Table 3). The ROI signal value of brain areas between the HR group and HCs are presented in Figure 3.

**Table 3:** Brain regions with significant difference in ALFF between HR patients and HCs.

**Brain areas**	**MNI coordinates**			**BA (aal)**	**Number of voxels**	***T* value**
	X	Y	Z			
HC > patient						
Frontal_Sup_Medial_L	0	57	18	23	595	−5.8697
Frontal_Mid_L	−39	6	57	7	325	−5.0923
HC < patient						
Cerebelum_8_L	−15	−60	−48	103	162	4.2122
Vermis_8	3	−72	−36	114	200	4.1573
Temporal_Inf_L	−54	−45	−24	89	222	5.2313
Cerebelum_6_R	42	−33	−30	100	153	4.3988

Notes: The statistical threshold was set at voxel with *P* < 0.01 for multiple comparisons using false discovery rate.

Abbreviations: ALFF: amplitude of low-frequency fluctuation; BA: Brodmann area; HR: hypertensive retinopathy; HC: healthy control; MNI: Montreal Neurological Institute.

## DISCUSSION

Rs-fMRI uses BOLD signal changes in local brain tissue to explore the overall or local brain activity and metabolism in a task-free state. ALFF reflects the correlation of neural activity between brain regions and information transmission characteristics, and communication under pathological or physiological conditions. Our research aimed to determine whether there were changes in ALFF values between HR and HCs patients’ brain regions. The rs-fMRI results showed that compared with HCs, HR patients in the LMSFG and LMFG had a decreased ALFF value. The left inferior cerebellar lobe, vermis, LITG, and right superior cerebellar lobe showed increased ALFF value in the HR group.

The middle frontal gyrus (MFG) occupies most of the frontal lobe between the superior frontal sulcus and participates in visual-spatial working memory [24] and attention control [25]. The posterior part of the MFG is the cortical side-viewing center, which controls the bilateral eyeball’s simultaneous contralateral gaze and head and neck rotation. When it is stimulated, both eyes look to the opposite side, with dilated eyelids and pupils, and the head rotates to the opposite side. When it is injured, both eyes are temporarily deflected to the affected side, and there is contralateral gaze paralysis. The early stages of blindness reduce functional connectivity between the temporal and frontal regions during motion processing [26]. In other words, blindness makes it difficult for the frontal lobe of the brain to be activated. It’s difficult for the patients to concentrate because of the impaired eyesight. Early studies showed that the middle frontal gyrus was damaged in patients with clinical depression [27–28]. Recent studies by Quinn et al. have certified that the MFG is indeed related to depression [29]. In our study, the ALFF value in the LMSFG of the HR group was lower than that of the HCs group, indicating frontal lobe injury in HR patients, which may be related to depression and reduced vision.

The medial section of the superior frontal gyrus (SFG), located in front of the paravertebral central lobule, passes forward through the cingulate gyrus [30]. It is mainly responsible for executive process and functions and is the basis of decision-making [31]. The medial frontal cortex is a portion of the default mode network (DMN). In the resting state, DMN is a network composed of brain regions that are still continuously performing certain functional activities in the non-task resting state of the brain, closely related to cognitive function and conscious state [32–33]. The DMN activity is almost unaffected when performing tasks that do not require cognitive involvement. However, when performing difficult cognitive tasks, related brain activities would affect the network’s activities. The more complex the cognition, the more pronounced the effect, and the two showed negative correlation. DMN was negatively activated when performing external tasks but positively activated when performing internal psychology tasks. The activation state is correlated to the task load [34]. In coping with the external environment’s dynamic changes, DMN integrates internal resources and monitors the external environment, which is more conducive to making decisions and plans and can better adapt to various environmental changes. Existing research shows that the DMN brain area is closely related to multiple psychiatric diseases such as depression and anxiety [35–36]. In the present study, the left SFG values were significantly lower in HR patients than in HCs, suggesting that HR may result in DMN damage. Therefore, it can be speculated that patients with HR may suffer from depressive symptoms due to damaged brain nodes of DMN.

The cerebellum is situated in the posterior fossa behind the medulla oblongata. It is an essential center of motor regulation, and it participates in coordinating autonomous movement and controlling balance [37]. For a long time, clinicians’ and researchers’ understanding of the cerebellum was often related to body balance, muscle stretching, and motor regulation. Nevertheless, with the rapid development of imaging technology in recent years, progress in cerebellar functional research has been extended to cognitive ability and emotional and visual processing [38]. More recent research indicates that the cerebellum may also be related to more advanced brain functions. A previously positron emission tomography (PET) imaging study showed that people with social anxiety disorder have abnormal signals in their cerebellum which increased blood flow to the brain, thus indicating that the cerebellum plays a vital role in the occurrence and development of anxiety and depression [39]. Our research show that the ALFF value increased significantly in the HR group’s cerebella, which means that cerebellar activity changes are the basis for depression in HR patients.

The cerebellum has a longitudinally narrow structure that curls up like a worm and is referred to as the “vermis” [18], which is the center of balance and motor control [40]. The vermis is closely related to the spinal cord and the vestibular nuclei, and if lesions occur, a balance disorder may result. Existing research shows that the cerebellum and posterior hemispheres’ vermis specifically activate eye and hand movement [41]. The lobular region of the cerebellar vermis is proprioception, which can recognize the relative position of body parts used for movement. This part of the cerebellum accomplishes proprioception by comparing movement commands from the brain with the spine’s sensations. Moreover, in patients with vision-guided saccade and convergence defects (a kind of systematic binocular vision disorder), the activity of vermis changes [42–43]. The increase of ALFF value in the HR group indicated more significant vermiform activity that may lead to the damage of HR patients’ motor control function.

The inferior temporal gyrus (ITG) is located below the middle temporal gyrus. The ITG is part of the ventral visual pathway that controls natural scene coding in the visual cortex and efficient neural coding for information processing [44]. In addition to this role, the ITG participates in advanced cognitive functions such as visual perception, cognitive language, and decision-making [45]. Furthermore, the ITG is a crucial area to promote cognitive processing and emotional regulation [12]. Early studies have found that ophthalmic diseases such as PAOG and blindness show dysfunction in this area [46, 47]. According to our research, the ALFF value of the left ITG was significantly higher in the HR group than in the HCs, manifesting the enhancement of ITG activity. Thus, we consider that HR patients might be associated with dorsal visual pathway dysfunction.

**Figure 4:**
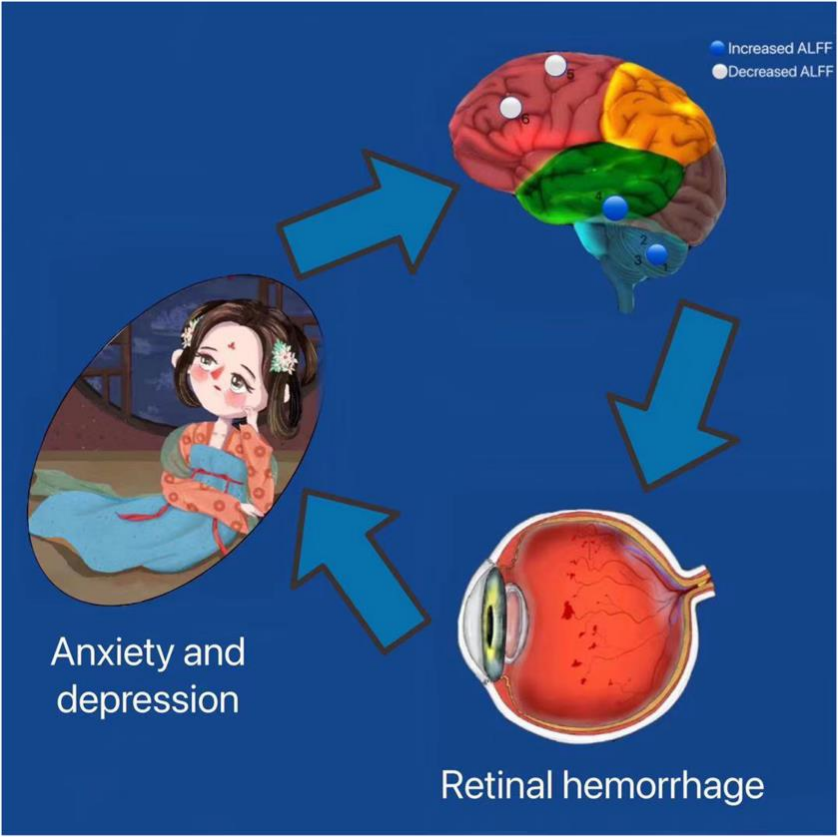
**Correlation between functional MRI and clinical manifestation and psychological emotion of HR patients.** The ALFF values of the medial LSFG and the LMFG in patients with HR were decreased. HR patients performed more anxiety and depression. Abbreviations: HR: hypertensive retinopathy.

Based on our above findings, we constructed a table and picture of the functions corresponding to various brain regions and the effects of alterations associated with HR on functional activities (Table 4 and Figure 4).

**Table 4:** Changes in specific brain regions and their potential functional effects.

**Brain regions**	**Experimental result**	**Brain function**	**Anticipated results**
Cerebellum posterior lobe	HRs > HCs	Motor control, cognitive, emotional and visual processing	Social and emotional problems, depression and anxiety
Vermis	HRs > HCs	Balance and motor control, oculomotor processing	Binocular visual impairment
Left inferior temporal gyrus	HRs > HCs	visual perception, cognitive language and memory	dysfunction of the dorsal visual pathway.
Left superior frontal gyrus, medial	HRs < HCs	Controls spontaneous eye movements, part of the default model network	Mental disorders, including depression and anxiety
Left middle frontal gyrus	HRs < HCs	working memory and attention control	Inattention, Depression and anxiety

Abbreviations: HR: hypertensive retinopathy; HC: healthy control.

### Summary and prospect

This study shows that the ALFF value of specific brain areas in HR patients fluctuated significantly. Changes in ALFF values reflect over- or reduced activity in brain regions. In recent years, research on the pathogenesis of metabolic encephalopathy by rs-fMRI has gradually increased. The research on hypertension-related cognitive dysfunction has become a hot spot. Abnormal ALFF values in these brain regions can predict early HR development and help prevent hypertensive microangiopathy malignant transformation. Significant progress has been made in the etiology, pathology, diagnosis, and treatment of hypertensive retinopathy, which has important guiding significance for clinical practice. However, further experimental and treatment-related investigations are needed to validate these results.
